# Different Spontaneous Pulmonary Metastasis Inhibitions against Lewis Lung Carcinoma in Mice by Bisdioxopiperazine Compounds of Different Treatment Schedules

**DOI:** 10.3797/scipharm.0910-16

**Published:** 2009-12-29

**Authors:** Da-Yong Lu, Fu-Geng Wu, Zu-Ming Zhen, Ting-Ren Lu, Hong-Ying Wu, Jin-Yu Che, Bin Xu

**Affiliations:** 1 Shanghai University, Shanghai 200444, PR China; 2 Shanghai Institute of Materia Medica, Chinese Academy of Sciences, Shanghai 201203, PR China; 3 College of Sciences, Shanghai University, Shanghai 200444, PR China

**Keywords:** Neoplasm metastasis, Bisdioxopiperazine compounds, Cancer chemotherapy, Probimane, Razoxane

## Abstract

Spontaneous neoplasm metastasis, a fatalist pathological feature of cancer, is a long-evolving, multi-steps process that can now only be treated or controlled by drugs or immuno-modulators. As we have previously hypothesized, each drug or immuno-modulator might act differently within various stages of a metastasis. Therefore any researches helping to determine these differences will be beneficial for updating therapeutics for metastasis. In this work, we have testified this hypothesis by using a series of well-known anti-metastatic agents – Bisdioxopiperazine compounds.

## Introduction

There have been two most difficult problems in cancer biology and therapeutics, neoplasm metastasis and multi-drug-resistances (MDR). Among these two thorny problems, treatments of neoplasm metastasis are especially difficulty and should be placed on higher agenda of the highest for its fatalist pathogenesis features and unpredictability of therapeutic outcome at the stage of drug initiation. Also, metastasized tumors often concomitantly manifest the characters of MDR. Therapeutic reports of metastasis treatment nowadays are overwhelmed with researches and applications of antivascular (angiogenesis) and MMPs inhibitors and more than 300 agents of different chemical formulae have been mentioned in the literatures [1–3]. Currently, all FDA licensed or internationally available anti-metastatic drugs share such characteristics and are regarded as a universal pathway for these drugs. However, these types of drugs are far from success in practical usage. Unfavorable reports are also enumerated such as indiscriminative molecular inhibition and generally low benefits for patients’ survival. More importantly, these therapies are also not without toxicity [4]. Unfortunately failures occur in most of the cases. Therefore how to optimistically use of these drugs in metastasis treatments remains to be a great challenge and any small breakthrough in this area will lead to great clinical achievements in cancer therapies.

Tumor metastases involve a fixed course of pathophysiological processes, and are responsible for more than 60% of cancer deaths worldwide [5–7]. Spontaneous neoplasm metastasis is a long pathogenesis process to deteriorate the hosts in natural conditions, at least a week-long course in mice and a month-long course in human. It encompasses at least three main different substages:

- tumor detachment from primary location;
- tumor cells flow in the blood or lymphatic vessels;
- tumor cell attachment and penetration through blood vessels of distant organs and angiogenesis [5].

We have previously hypothesized that many anti-cancer or anti-metastatic drugs might act differently in the different courses of these substages [8]. In order to testify this hypothesis, we carried out an experiment to compare the inhibition of drugs against an established model, Lewis lung carcinoma (3LL), for observing spontaneous metastasis inhibitions using different therapeutic protocols.

**Fig. 1.**
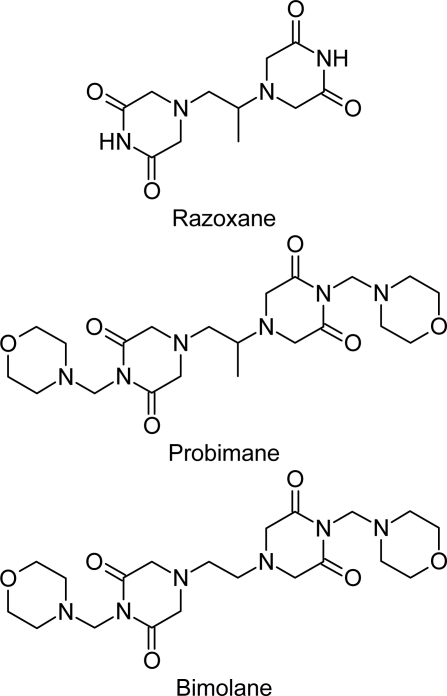
The structural formulae of three bisdioxopiperazine compounds

Bisdioxopiperazine compounds (Biz), including ICRF-154, Razoxane (ICRF-159, *Raz*), ICRF-186 and ICRF-187 (two stereo-isomers of Raz) and ICRF-193, developed in the UK, have been a series of serendipitous agents found to be effective against a model of spontaneous metastasis (Lewis lung carcinoma, 3LL) [9, 10]. Ever since their development in 1969, many studies have addressed their potential use and mechanisms of action. Since Biz compounds are unique and conservative in pharmacological actions, their new analogs Probimane [4,4′-propane-1,2-diylbis[1-(morpholin-4-ylmethyl)piperazine-2,6-dione]; AT-2153, Pro] [7–10] and Bimolane [4,4′-ethane-1,2-diylbis[1-(morpholin-4-ylmethyl)piperazine-2,6-dione]; AT-1727, Bim] [11–16] were synthesized at the Shanghai Institute of Materia Medica, Chinese Academy of Sciences, Shanghai, China. The structural formula of these three Biz analogs is represented in Figure 1.

The researches of anti-metastatic drugs, though at the odds nowadays facing, will be the hottest spot and discipline in the future. This work is among this very phase of great transitions of understanding and knowledge about metastasis treatments from drug discovery only into clinical drug options in combinations with.

## Materials and methods

Drugs and Reagents: Pro, Bim and Raz were prepared by Prof Yun-Feng Ren at the Department of Medicinal Chemistry, Shanghai Institute of Materia Medica, Chinese Academy of Science and suspended in 5 g/L carboxymethylcelluose (CMC) solution before use.

Animals and Tumor Models: C57BL/6j strain mice were purchased from Shanghai Center of Laboratory Animal Breeding, Chinese Academy of Sciences and were conducted experimentations in compliance with the Guidelines for the Care and Use of Research Animals, NIH, established by Washington University’s Animal Studies Committee.

Neoplasm Metastases Determination: Since anti-metastatic therapeutic would be rather preventive than indiscrimination cytotoxic against normal and neoplasm tissues, we used a low dosage of Biz compounds (1/10 to 1/5 LD_50_) to basically offset a possible lethal or mutational toxicity of conventional therapies involved in such experimental study. Biological inoculation with pulmonary nodules of 3LL was a method used by Yue et al [14]. C57BL/6j mice were implanted sc with LLC (2×10^6^ cells) from donor mice. The experiment was undergone in two different sets of schedules for day2 and day8 initiation of therapy. Either from day2 or day8 after tumor inoculation, the mice were injected ip with drugs daily in 10 injections. On day 18, all mice were sacrificed, and locally grown tumors were separated from skin and muscles and weighed. The 10 lungs of 5 host mice of each group were sterilely homogenized with sterilized normal saline (2 ml). These homogenized lungs were separately injected sc into 10 healthy C57BL/6j mice. The numbers of mice with newly locally grown tumors were counted after 1 month later.

### Statistics

Samples were subjected to statistical analysis for distinguishing between treated and control.

## Results

We used the doses of 1/10 and 1/5 LD_50_ (from the unpublished experimental data of Shanghai Institute of Materia Medica, Chinese Academy of Sciences) in the following therapeutic experiment.

The Effects of Biz on Pulmonary Metastasis of 3LL: Table 1 showed that Pro and Bim significantly inhibited the pulmonary metastasis of 3LL both following day2 and day8 injections, but Raz only significantly inhibited the pulmonary metastasis of 3LL following day2 injections. Pro inhibited the pulmonary metastasis of 3LL more potently than Bim did at equitoxic dosage. Comparatively, it seems that Pro has superior inhibition of pulmonary metastasis of 3LL than Bim and Raz for its exclusive targeting potential.

## Discussion

Since tumor metastasis is responsible for more than 60% cancer death worldwide, metastasis therapy, as a result, remains to be a biggest challenge to all cancer therapeutics. Biz compounds are well-known for their anti-metastasis action. However, their modes of action and therapeutic profile remain to be investigated for their potential clinical availability. The research work for data has been mostly focusing on anti-angiogenesis action [18–20] and others of sialic acids or fibrinogen-pathway inhibitions [21, 22]. Different inhibitory characteristics of the three Biz compounds, Bim, Raz and Pro to pulmonary metastasis of 3LL were evidences from the results of this experiment. The major difference between Raz and Pro is that Raz treated day8 after the tumor inoculation showed simply no inhibition on pulmonary metastasis of 3LL. In contrary, Pro or Bim may have a wider therapeutic maintenance than Raz. It suggests that the morpholine group presented in structures may play a role for their potential treatments of formed metastatic foci. Two previous data of our experimentations can support and explain this evidence of great divergence of metastasis inhibition between different Biz compounds and treatment schedules. Pro has a better therapeutic index than Bim although they may act through same pathways and have similar molecular targets. The development of pathogeneses of tumor metastasis has be categorized further into three critical steps; (i) tumor detachment from primary location; (ii) tumor cells flow in the blood or lymphatic vessels; (iii) tumor cell attachment and penetration through blood vessels of distant organs. From the report of James et al, the detachment of 3LL began at day 6–8 [23]. The inhibition of neoplasm metastasis by Raz has been previously explained as a result of reducing in the formation of blood vessels in tumor tissues (neovasculature) [18–20]. This study further supports this mechanism by reiterating with targeting for tumor cell detachment from primary sites. Thus, it has been proposed that Raz remains ineffective against the pulmonary metastases of 3LL (formed metastatic foci). This data can be used to explain also why Raz was reported to be more effective against neoplasm metastases for spontaneous metastatic tumors rather than for artificial ones [2]. However, Pro and Bim might be equally effective in both the cases. From our early data of ^14^C-probimane tracing and autoradiography [24, 25], an obvious greater accumulation of Pro was found in tumor tissues, especially in metastatic foci. It can help to explain that Pro can more effectively inhibition of neoplasm metastasis than Raz in formed metastatic foci through stronger antiproliferative effect [13, 14]. Their molecular mechanisms of action may be attributed to their inhibition of CaM-activated Ca^++^-Mg^++^-ATPase [26].

This experimental evidence further supports our hypothesis that each drug or immuno-modulators might act differently within various stages of a metastatic course. In general, we propose that the MMPs inhibitors might be more active in preventing tumor cells from detaching from primary locations. Immuno-modulators might promote the activity of macrophages in killing of tumor cells in vascular and lymphatic circulation [27]. However, highly cytotoxicity agents, alone or in combination with angiogenesis inhibitors might be more effective in treatment of formed metastatic foci. It helps us to better use the drugs in treating cancer metastases in the future.

## Figures and Tables

**Tab. 1.** The effects of Biz on pulmonary metastasis of Lewis lung carcinoma.

**Compounds**	**Dosage ip×10**	**Initial treated/d**	**PTI%^a^**	**MNMCST^b^**
Control	–	–	–	9.5±0.6
Probimane	12	2	18.6	1.5±0.2**
Probimane	24	2	20.3	1.0±0.2**
Probimane	12	8	19.2	1.5±0.2**
Probimane	24	8	21.0	1.0±0.0**
Bimolane	6.5	2	18.3	3.5±0.2*
Bimolane	13	2	20.1	3.5±0.2*
Bimolane	6.5	8	13.1	4.0±0.4*
Bimolane	13	8	18.2	4.0±0.4*
Razoxane	6.5	2	19.2	1.5±0.2**
Razoxane	13	2	22.0	1.5±0.2**
Razoxane	6.5	8	15.0	9.0±0.0
Razoxane	13	8	15.8	8.5±0.8

Ten mice were used for each group, and each group was repeated once;

* P<0.05;

** P<0.01;

a PTI … primary tumor inhibition;

b MNMCST … mean number of mice carrying secondary tumors
